# Inclusion of juvenile stages improves diversity assessment and adds to our understanding of mite ecology – A case study from mires in Norway

**DOI:** 10.1002/ece3.9530

**Published:** 2022-12-12

**Authors:** Anna Seniczak, Stanisław Seniczak, J. Carlos Iturrondobeitia, Martyna Marciniak, Sławomir Kaczmarek, Joanna Mąkol, Andrzej Kaźmierski, Andrzej Zawal, Marla D. Schwarzfeld, Kjell Ivar Flatberg

**Affiliations:** ^1^ Department of Natural History University Museum of Bergen, University of Bergen Bergen Norway; ^2^ Department of Evolutionary Biology, Faculty of Biological Science Kazimierz Wielki University Bydgoszcz Poland; ^3^ Department of Zoology and Cellular Animal Biology University of the Basque Country Leioa Bizkaia Spain; ^4^ Department of Animal Morphology Adam Mickiewicz University in Poznań Poznań Poland; ^5^ Department of Invertebrate Systematics and Ecology Wrocław University of Environmental and Life Sciences Wrocław Poland; ^6^ Institute of Marine and Environmental Sciences, Center of Molecular Biology and Biotechnology University of Szczecin Szczecin Poland; ^7^ Canadian National Collection of Insects Arachnids and Nematodes Ottawa Ontario Canada; ^8^ NTNU University Museum, Norwegian University of Science and Technology Trondheim Norway

**Keywords:** Mesostigmata, Trombidiformes, Sarcoptiformes, Oribatida, *Sphagnum*, peatlands

## Abstract

Arachnid orders, Mesostigmata, Trombidiformes, and Sarcoptiformes, commonly known as ‘mites’, are abundant in mires, both as adults and as juveniles. However, due to the challenges of identification, the juvenile forms are often excluded from analyses. This is the first study in mires that included all three mite orders identified to the species level, including juvenile instars. We aimed to compare how diversity and the response to ecological variables differed if only the adults (ad) vs. the total number of specimens (ad+juv) are considered. Samples of 20 *Sphagnum* species (five subgenera) were collected and mites were extracted using Berlese funnels. Overall, nearly 60,000 mites were analyzed; of these Mesostigmata made up 1.87% of the total, Trombidiformes −0.27%, and Sarcoptiformes −97.86%. The study revealed 154 species (33 Mesostigmata, 24 Trombidiformes, and 97 Sarcoptiformes), the highest diversity of mites ever reported from mires. The inclusion of juveniles increased observed species richness by 6%, with 10 species (one Mesostigmata, six Trombidiformes, and three Sarcoptiformes) represented only by juvenile forms. Seventeen species are new to Norway (four Mesostigmata, one Sarcoptiformes, and 12 Trombidiformes, including five undescribed species of Stigmaeidae and Cunaxidae). Four of these were represented in the samples only by juveniles. Including the juveniles explained a greater amount of the variability of Trombidiformes (explanatory variables account for 23.60% for ad, and 73.74% for ad+juv) and Mesostigmata (29.23% − ad, 52.91% − ad+juv), but had less of an impact for Sarcoptiformes (38.48% − ad, 39.26% − ad+juv). Locality, *Sphagnum* subgenus and species, wetness, and trophic state significantly affected the mite communities and should be taken into consideration when studying mires. Since juvenile stages contribute significantly to mite diversity in mires, they should also be included in mite studies in other habitats.

## Graphical abstract

Including the juveniles explained a greater amount of the variability of Trombidiformes and Mesostigmata, but had less of an impact for Sarcoptiformes. Including juveniles increased the observed species richness by 6% in comparison to data based on adults only. The study revealed the highest diversity of mites (154 species) ever reported from mires, including five species new to science and twelve new to Norway.

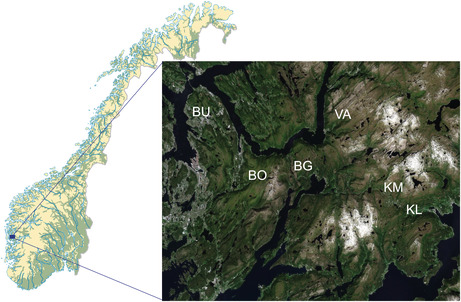

## 1 INTRODUCTION

Peatlands, including mires (i.e., living peatlands that can accumulate peat) host highly specialized and unique flora and fauna, due to their nutrient‐poor, acidic, and water‐saturated conditions. As such, they make a significant contribution to global biodiversity. They store twice as much carbon as all the world's forests, which is of paramount importance in relation to climate change, in particular temperature increase and changes in precipitation (Parish et al., 2008). They are also important water regulators, as they, like huge sponges, accumulate water during wet seasons and release it during dry seasons (Joosten et al., 2017). In Norway, peatlands cover 13.8% of the country and are the best preserved in Europe (Tanneberger et al., 2017). About 85% of them are still able to accumulate peat, while the average value in Europe is 50%, and in many countries, it is even lower (e.g., Finland 40%, Ireland 18%, Poland 16%, and Germany 2%) (Joosten et al., 2017). Classification of mires varies in European countries according to each country's traditions (Joosten et al., 2017). In Norway, the common practice is to classify mires according to hydromorphological patterns (mire massif types, Moen in Joosten et al., 2017 p. 540) and vegetation types (Moen in Joosten et al., 2017 p. 18), and without formal phytosociological classification. The main vegetational classification divides mires into ombrotrophic mires (bogs) fed by precipitation nutrients only, and minerotrophic mires (fens) fed by an additional supply of mineral soil nutrients from the mineral soil surroundings. The classification pattern found on local mire sites is primarily based on the variation in vegetation along two eco‐gradients, (1) the ‘poor‐rich‘ gradient, reflecting increased nutrient conditions of the mire habitat (trophic gradient) and (2) the ‘dry‐wet‘ gradient, reflecting increased wetness conditions of the mire habitat (wetness gradient).

Mires are considered species‐poor habitats (Rydin & Jeglum, 2006), but some groups of organisms, in particular *Sphagnum* mosses (Laine et al., 2018) and mites (Vilkamaa, 1981), are abundant and highly diverse within them. Norwegian mires have the most species‐rich peat moss flora in Europe, with 54 named species, that is, 90% of all known European species (Laine et al., 2018). They also host the highest diversity of the oribatid mites (a group within Sarcoptiformes) ever found in this type of habitat, largely explained by the large *Sphagnum* diversity (Seniczak & Seniczak, 2020). In limited sampling in Norwegian mires, up to 95 species of Oribatida were found (i.e., 30% of known species in Norway), which is comparable to the diversity of this group in broadleaf forests (Seniczak, Niedbała, Iturrondobeitia, et al., 2021; Seniczak, Seniczak, Graczyk, et al., 2021; Seniczak, Seniczak, Starý, et al., 2021). These included 18 new records for Norway and two species new to science (Seniczak & Seniczak, 2020, 2021). The latter demonstrates that the potential of mires for the protection of species diversity associated with this specific habitat is still underestimated.

Mesostigmata, Trombidiformes, and Sarcoptiformes are small arachnids; most species are below 0.5 mm in length, with only Parasitengonina reaching larger sizes (usually 2–4 mm). They are commonly called ‘mites’, although they belong to two separate superorders: Parasitiformes (Mesostigmata) and Acariformes (Trombidiformes and Sarcoptiformes). They are primarily terrestrial animals, but some are adapted to amphibious or even aquatic habitats and can be abundant in mires (Gerecke et al., 2016; Schatz & Behan‐Pelletier, 2008; Walter & Proctor, 2013). Most Mesostigmata are predators (primarily feeding on nematodes and microarthropods, including soft‐bodied mites), although there are also some species that feed on fungi, pollen, algae, and bacteria or parasites (Lindquist et al., 2009b; Seniczak, Graczyk, et al., 2018; Walter & Proctor, 2013). Trombidiformes are extremely diverse, with various feeding preferences (algivores, bacterivores, fungivores, herbivores, predators, and parasites), while Sarcoptiformes are mostly saprophagous (Walter et al., 2009; Walter & Proctor, 2013).

**FIGURE 1:**
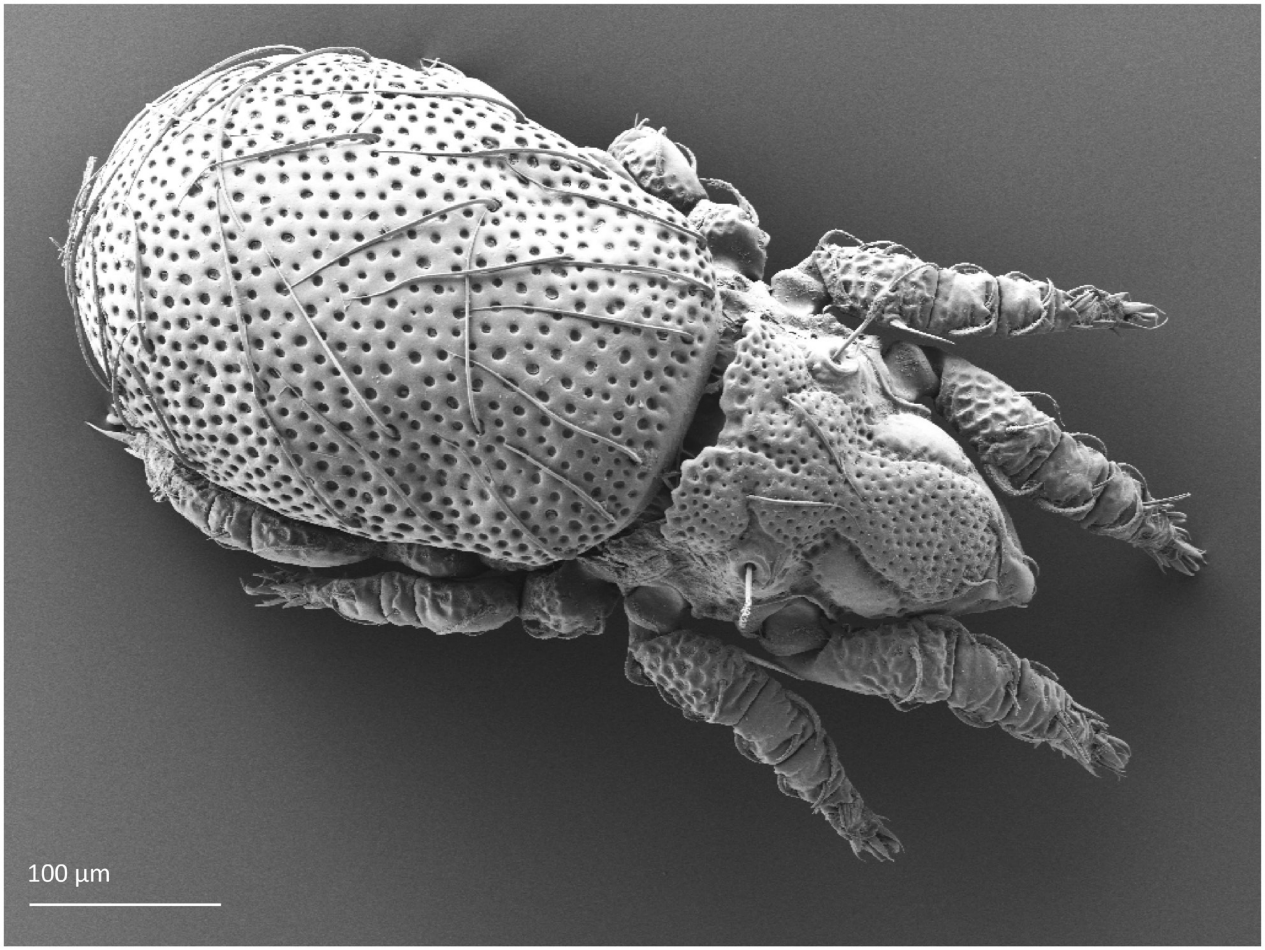
*Nanhermannia coronata* (Sarcoptiformes, Oribatida), adult; the most abundant mite species collected in this study.

While the knowledge of Oribatida (Figure 1), the largest group of Sarcoptiformes, in mires has been constantly improving in recent decades (summarized by Mumladze et al., 2013; Seniczak, 2011), relatively few, scattered data are available about Mesostigmata (Bolger, Arroyo, & Piotrowska, 2018; Kaczmarek et al., 2006, 2011; Kaczmarek & Marquardt, 2007, 2008; Salmane & Spuņģis, 2015; Skorupski et al., 2008; Wisdom et al., 2011). Trombidiformes of mires (except for Hydrachnidia) are even less well known (Philippov et al., 2021; Willmann, 1933).

The vast majority of studies on mites in peatlands have only identified adults to species (Barreto et al., 2021; Donaldson, 1996; Lehmitz, 2014; Lehmitz et al., 2020; Markkula, 2014; Markkula et al., 2018, 2019; Minor et al., 2016, 2019; Philippov et al., 2021; Solhøy, 1979). Some studies, however, indicated the importance of including juvenile instars. For example, in a study on the effect of warming on oribatid mites, significant differences in the ratio of immature stages to adults were observed in different types of fens (Barreto et al., 2021). In Norwegian mires, juveniles constituted nearly 40% of oribatids; in some species, they highly dominated the age structure (up to 80% in *Nothrus* spp.), and two oribatid species were represented only by juveniles (Seniczak, Seniczak, Iturrondobeitia, et al., 2020). Similarly, juvenile forms can be important for answering ecological questions in other ecosystems, such as forests (Proctor et al., 2002). For example, the ratio of adults to juveniles can be a useful measure of forest habitat disturbance (Maaroufi et al., 2022).

The postembryonic development of mites includes several instars. The full development that occurs in Sarcoptiformes and many Trombidiformes includes a hexapod (i.e., three‐legged) larva, three octopod (four‐legged) nymphal stages (protonymph, deutonymph, and tritonymph) and adult. In some groups (e.g., Mesostigmata and some Prostigmata within Trombidiformes), the number of instars is reduced (Walter & Krantz, 2009).

Identification of juvenile stages is laborious and often more challenging compared to adults, due to their smaller body size, more delicate structures, and above all, because for many mite species these forms remain unknown (Solhøy, 1979). For example, among Oribatida, the juveniles of only about 8% of the known oribatid mite species and 30% of genera have been described (Norton & Ermilov, 2014). For mesostigmatid mites, it is difficult to assess the percentage of species with full ontogeny known; however, in most taxa, only adults are described. In some species groups, there are also taxa known only as juveniles; for example, in the *Trichouropoda ovalis* group, Hirschmann and Wiśniewski (1986) described 45 species, and about half of them (53%) are only known as deutonymphs. Among Trombidiformes the situation varies. In terrestrial Parasitengonina (Calyptostomatoidea, Erythraeoidea, and Trombidioidea), the proportions are reversed due to greater interest in parasitic larvae, particularly chiggers (Mąkol & Wohltmann, 2013); moreover, the heteromorphism of active instars resulted in independent descriptions of species known from larvae and from active postlarval forms. Of the approximately 5000 species assigned to this group, almost 80% have only been described as juveniles, and the share has been largely influenced by vertebrate‐associated chiggers (Mąkol & Wohltmann, 2013). For an array of species, only the reference to the postlarval form (without distinction between juvenile deutonymph and adult form) has been provided in the literature. Among the water mites (Hydrachnidia) of the northern hemisphere, juvenile stages are known for almost all genera, but probably for <10% of the species (Prasad & Cook, 1972; Tuzovsky, 1987, 2011; Wainstein, 1976, 1980; Zawal, 2008). Among the other Prostigmata, the state of knowledge varies considerably between taxa; however, the majority of species descriptions are based on the adult instars, with juveniles included more haphazardly, depending on the taxonomist and the specimens available.

In Mesostigmata, only two nymphal stages are present (protonymph and deutonymph) and the tritonymph is lacking. However, in some earlier works, because of differences between young (smaller) and old (larger) protonymphs, they were described as protonymphs and deutonymphs, respectively, thus describing the real deutonymphs as tritonymphs (Harris, 1973; Lindquist et al., 2009b; Marquardt & Kaczmarek, 2017, 2019; Walter & Proctor, 2013; Womersley, 1960). Feeding habits of most Mesostigmata are only partially known and usually juveniles coexist with adults. In some Mesostigmata (e.g., Uropodina and Sejida), deutonymphs are phoretic, that is, they are transported by arthropod hosts (e.g., insects or myriapods) while attached to their host's body by a pedicellar stalk (Bajerlein et al., 2013; Bajerlein & Przewoźny, 2012; Bajerlein & Witaliński, 2012, 2014; Hirschmann et al., 1991).

Within Trombidiformes, Parasitengonina (including both terrestrial species and Hydrachnidia) have a very complex life cycle with only some stages being active (larvae, deutonymphs, and adults) while the protonymph and tritonymph are calyptostatic, that is, nonfeeding and non‐motile forms (Grandjean, 1938). Within the active stages, the larvae have very different feeding habits from the predaceous deutonymphs and adults, being generally parasitic on arthropods or vertebrates. Due to their parasitic habits, often with flying insects as hosts, these larvae are underrepresented compared to deutonymphs and adults in samples extracted in Berlese or Tullgren funnels (e.g., Wohltmann et al., 2006). Most other Prostigmata have less complex life cycles, with immature stages resembling adults both morphologically and ecologically, and co‐existing with adult populations. The most extreme change is from hexapod larva to octopod nymph, with the remaining changes pertaining to increasing complexity (e.g., increasing setal counts and increasing number of genital papillae; Walter et al., 2009).

In most Sarcoptiformes, the juveniles co‐occur in the same microhabitat as adults (although they may occupy different ecological niches by having different food preferences). But, for example, in ptyctimous mites, the juveniles live in galleries inside dead wood or conifer needles and cannot be extracted with simple methods like Berlese or Tullgren apparatus (Hågvar, 1998; Niedbała, 1992).

In the present study, three mite orders (Mesostigmata, Trombidiformes, and Sarcoptiformes) in peatlands are investigated for the first time at the species level, including both adult and juvenile forms. The aim of this paper is to compare how species diversity (measured by species richness) and ecological patterns are affected if only the adults (ad) vs. the total number (ad+juv) are considered. Because juveniles are found abundantly in mires, we hypothesized that including juveniles will i) result in discovery of the higher species diversity of all mite groups, and ii) improve our understanding of the effect of ecological factors on the variability of mite communities.

## 2 MATERIALS AND METHODS

### 2.1 Sampling and material analysis

**FIGURE 2:**
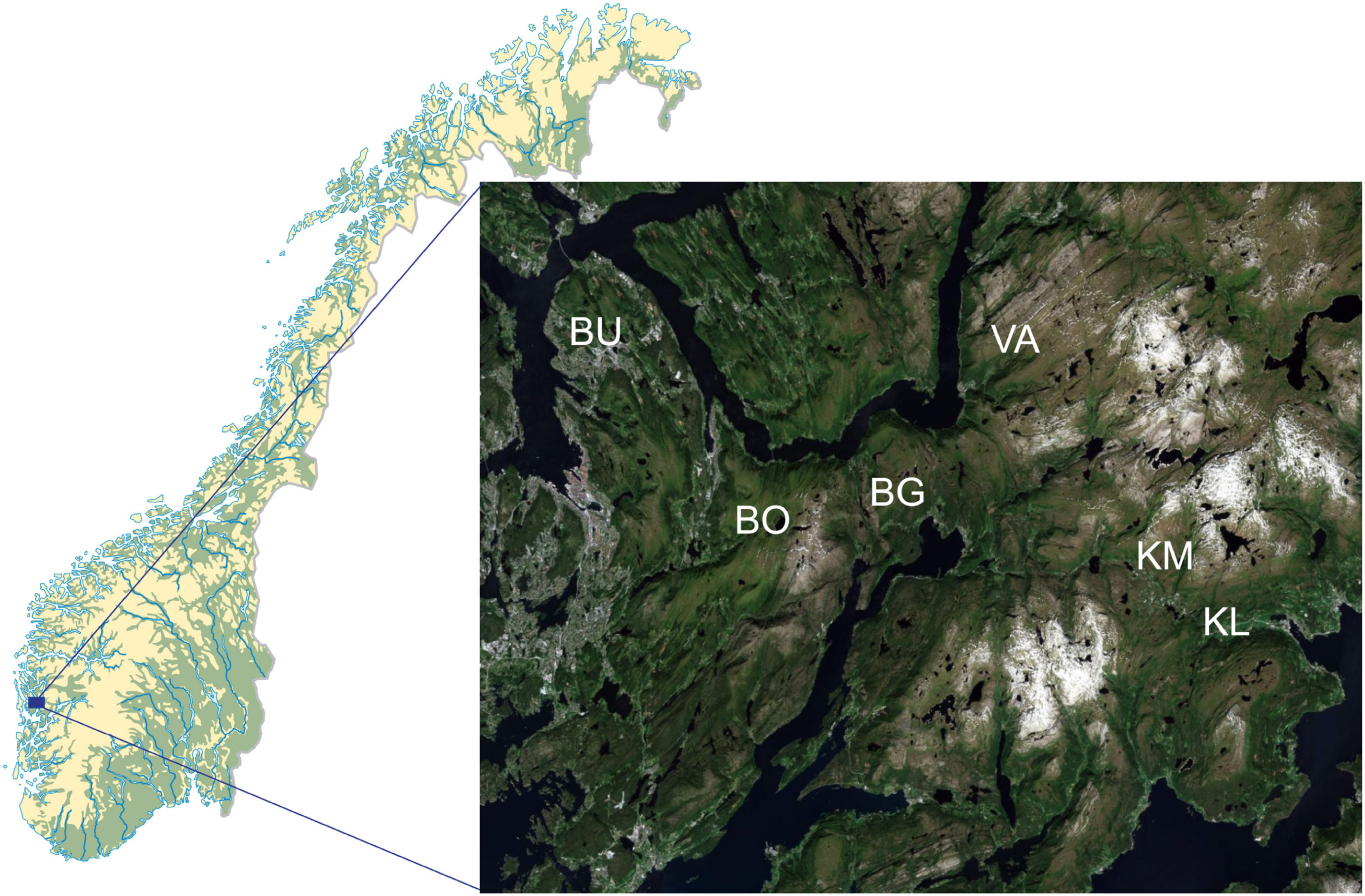
Location of the studied mires in Norway (modified from https://www.norgeskart.no); detailed information about the sampling locations is in Table 1.

The sampling was carried out in six mires located in the western, oceanic part of Norway (Table 1, Figure 2). The climate of the region is mild and relatively warm (the average annual temperature is 6.8°C) and has high precipitation (annual rainfall is 2251 mm). Climatic data were taken from Norwegian Centre for Climate Services (2022) (available at https://seklima.met.no/).

**TABLE 1:** Sampling design in mires of western Norway

Site	Location	Sample	*Sphagnum* subgenus	*Sphagnum* species	Trophic state	Wetness
BG	Bergen, Gullbotn (60.412, 5.642, 250 m a.s.l.)	1	*Sphagnum*	*S. centrale* C.E.O. Jensen	Intermediate	Hummock
		2	*Sphagnum*	*S. papillosum* Lindb.	Poor	Lawn
		3	*Acutifolia*	*S. subnitens* Russow et Warnst.	Intermediate	Lawn
		4	*Acutifolia*	*S. warnstorfii* Russow	Moderately rich	Lawn
		5	*Subsecunda*	*S. contortum* Schultz	Moderately rich	Lawn
KM	Kvam, Måvotsvatnet (60.393, 5.948, 440 m a.s.l.)	6	*Sphagnum*	*S. affine* Renauld et Cardot	Intermediate	Hummock
		7	*Sphagnum*	*S. divinum* Flatberg et Hassel	Poor	Lawn
		8	*Sphagnum*	*S. papillosum* Lindb.	Poor	Lawn
		9	*Acutifolia*	*S. capillifolium* (Braithw.) Warnst.	Poor	Hummock
		10	*Acutifolia*	*S. rubellum* Wilson	Poor	Lawn
		11	*Cuspidata*	*S. angustifolium* (C.E.O. Jensen ex Russow) C.E.O. Jensen	Na^a^	Na^a^
		12	*Cuspidata*	*S. fallax* (H. Klinggr.) H. Klinggr.	Poor	Lawn
		13	*Cuspidata*	*S. majus* (Russow) C.E.O. Jensen	Poor	Carpet
		14	*Cuspidata*	*S. pulchrum* (Lindb. ex Braithw.) Warnst.	Poor	Carpet
		15	*Cuspidata*	*S. tenellum* (Brid.) Pers. ex Brid.	Poor	Lawn
KL	Kvam, Langvotnevatnet (60.371, 6.024, 360 m a.s.l.)	16	*Cuspidata*	*S. pulchrum* (Lindb. ex Braithw.) Warnst.	Poor	Carpet
		17	*Cuspidata*	*S. riparium* Ångstr.	Poor	Carpet
BO	Bergen, Osavatnet (60.383, 5.539, 340 m a.s.l.)	18	*Sphagnum*	*S. affine* Renauld et Cardot	Poor	Lawn
		19	*Cuspidata*	*S. flexuosum* Dozy et Molk.	Poor	Lawn
VA	Vaksdal (60.482, 5.807, 400 m a.s.l.)	20	*Rigida*	*S. compactum* Lam. et DC.	Na^a^	Na^a^
		21	*Rigida*	*S. strictum* Sull.	Na^a^	Na^a^
		22	*Acutifolia*	*S. girgensohnii* Russow	Poor	Lawn
		23	*Acutifolia*	*S. molle* Sull.	Na^a^	Na^a^
		24	*Cuspidata*	*S. flexuosum* Dozy et Molk.	Poor	Lawn
BU	Bergen, Ulsetstemma (60.478, 5.308, 130 m a.s.l.)	25	*Rigida*	*S. strictum* Sull.	Poor	Carpet
		26	*Cuspidata*	*S. fallax* (H. Klinggr.) H. Klinggr.	Poor	Carpet
		27	*Cuspidata*	*S. riparium* Ångstr.	Poor	Carpet

Abbreviations: Na, not applicable.

^a^ Wet heath.

We selected sampling plots, each dominated by a single *Sphagnum* species, and collected 27 homogeneous samples of 20 cm × 20 cm and 5 cm deep, on 25–26 June 2008. Most of the samples originated from true mires (Table 1), and four were from Northern Atlantic wet heath, which is a slightly different habitat, although it often forms peat. *Sphagnum* mosses were identified to species classified into five subgenera: *Sphagnum* (four species), *Rigida* (two species), *Cuspidata* (seven species), *Subsecunda* (one species), and *Acutifolia* (six species). We aimed to collect as many different *Sphagnum* species as we could find; equal numbers of samples were not collected at each mire since homogeneous samples of the desired size were not always present. The nomenclature of *Sphagnum* spp. with author citations of species, as well as the subordinate classification in subgenera instead of sections, follow Laine et al. (2018).

In each of the 27 sampling plots, plant species within a 1 m × 1 m plot were identified. Based on floristic composition and relative abundance of indicator species, the samples collected in true mires were assigned to trophic state (poor, intermediate, and moderately rich) and wetness gradients: hummocks (mounds of peat), lawns (firm turf‐like vegetation), and carpets (softer than lawns, including quaking mats). Samples collected in wet heath were excluded from the analyses of the effect of trophic state and wetness gradients. Vegetation data are included in Appendix S1.

Samples were transported in plastic bags to the laboratory at the University of Bergen, stored at 4°C until the next day, and extracted using Berlese funnels for 2 weeks into 70% ethanol; the temperature above the sample was approximately 30°C, and additional ethanol was added as needed due to evaporation. All active life forms of mites that were obtained during the extraction were identified, including adults and juvenile instars, that is, larvae and nymphs.

Oribatida were temporarily mounted on cavity slides in lactic acid and adult specimens were identified using the key of Weigmann (2006), and the keys on juveniles scattered between several publications (Ermilov & Łochyńska, 2008; Pfingstl & Krisper, 2011; Seniczak, 1972a, 1972b, 1978a, 1978b, 1980a, 1980b, 1988, 1993; Seniczak et al., 1998, 2007, 2009; Seniczak & Klimek, 1990; Seniczak & Seniczak, 2007, 2009b, 2009c, 2012, 2020; Seniczak, Seniczak, et al., 2018; Seniczak et al., 2019; Seniczak & Żelazna, 1992; Shaldybina, 1986; Willmann, 1931). Family names follow Norton and Ermilov (2014), and species nomenclature follows Subías (2004, 2022), Weigmann (2006), Siepel et al. (2009), and Norton and Ermilov (2014). Endeostigmata were also mounted on temporary cavity slides in lactic acid and identified using Walter et al. (2009).

Mesostigmata and non‐parasitengonine Prostigmata were mounted on permanent slides, Mesostigmata in PVA mounting medium (Lactic Acid, Poly Vinyl Acetate, and Phenol Solution, BioQuip Products, Inc., Compton, CA, USA), and non‐parasitengonine Prostigmata in Berlese's mounting medium (a highly concentrated solution of chloral hydrate and gum arabic in water). Mesostigmata were identified following Bhattacharyya (1963), Błaszak (1974), Denmark and Muma (1989), Farrier (1957), Ghiljarov and Bregetova (1977), Gwiazdowicz (2007), Halašková (1970), Hyatt (1980), Hyatt and Emberson (1988), Micherdziński (1969), Kalúz and Fenďa (2005), Karg (1993), Mašán (2001, 2003), Mašán and Fenďa (2004). Prostigmata were identified using keys (Da Silva et al., 2016; Kaźmierski, 1998; Skvarla et al., 2014; Zacharda, 1980) and based on the taxonomic experience of A. Kaźmierski.

Terrestrial Parasitengonina were cleared in KOH and mounted on microscopic slides using Faure's fluid. For identification, we followed Wohltmann et al. (2006) and Łaydanowicz and Mąkol (2010). Adults of Hydrachnidia were dissected, slide mounted in Hoyer's medium, and identified by keys of Di Sabatino et al. (2010) and Gerecke et al. (2016). The classification of higher systematic categories follows Lindquist et al. (2009a).

Full names of the mites are given in Table 2 and Appendix S1, while in the figures, abbreviations are used. The new records of Mesostigmata for Norway refer to the most recent checklist (Gwiazdowicz & Gulvik, 2005a) and later references (Bolger, Devlin, & Seniczak, 2018; Castilho et al., 2015; Gwiazdowicz et al., 2013; Gwiazdowicz & Gulvik, 2005b, 2007; Kaczmarek et al., 2021; Kvifte et al., 2022; Neves Esteca et al., 2020; Seniczak, Bolger, Roth, et al., 2019; Seniczak, Niedbała, Iturrondobeitia, et al., 2021; Seniczak, Seniczak, Graczyk, et al., 2021; Seniczak, Seniczak, Iturrondobeitia, et al., 2020; Seniczak, Seniczak, Schwarzfeld, et al., 2020; Seniczak, Seniczak, Starý, et al., 2021; Słomian et al., 2005; Thunes et al., 2021; Venancio et al., 2016) while those of Trombidiformes are based on the checklist of Mehl (1979) and Stålstedt et al. (2019). The new records of Sarcoptiformes for Norway are based on the checklists of Mehl (1979) and Seniczak, Seniczak, Iturrondobeitia, et al. (2020); Seniczak, Seniczak, Schwarzfeld, et al. (2020).

**TABLE 2:** New species records for Norway; ad – adults, juv – juveniles; for site abbreviations see Table 1

Order/suborder and family	Species	Number	Site
Mesostigmata			
Ascidae	*Cheiroseius kargi* Gwiazdowicz, 2002	1 ad	BO
Laelapidae	*Ololaelaps sellnicki* Bregietova et Koroleva, 1964	35 ad, 14 juv	BG, KM, VA, BU
Phytoseiidae	*Amblyseius silvestris* Denmark et Muma, 1989	3 ad	BO, VA
Urodinychidae	*Dinychus kaluzi* Mašán, 1999	4 ad	BG
Trombidiformes			
Stigmaeidae	*Villersiella quadriscutata* Willmann, 1953	1 juv	VA
	*Cheylostigmaeus* sp. nov. I	14 ad, 7 juv	BO
	*Cheylostigmaeus* sp. nov. II	1 ad	KM
	*Stigmaeus rhodomelas* Berlese, 1910	1 ad, 1 juv	VA
	*Stigmaeus* sp. nov. I	13 ad, 5 juv	KM, BO, VA,
	*Stigmaeus* sp. nov. II	1 ad	VA
Cunaxidae	*Dactyloscirus* sp. nov.	1 ad	BO
Rhagididae	*Rhagidia gigas* (Canestrini, 1886)	3 juv	BG, KM
	*R. ruseki* Zacharda, 1980	1 juv	VA
	*Robustocheles montana* Zacharda, 1980	5 ad, 10 juv	KM, VA
	*R. mucronata* (Willmann, 1936)	3 juv	KM, BO
Microtrombidiidae	*Valgothrombium valgum* (George, 1809)	2 ad, 3 juv	BG
Sarcoptiformes			
Endeostigmata			
Alycidae	*Bimichaelia diatema* Grandjean, 1939	2 ad	KM, VA

### 2.2 Statistical analyses

The statistical analyses were based on the abundances of adults and juveniles in each mite group. All multivariate analyses were performed using CANOCO software (Microcomputer Power, Ithaca, NY, USA; Jongman et al., 1995; Ter Braak, 1988). Response data (biological species) were log‐transformed, log (*x* + 1) (Łomnicki, 2017) in order to down‐weight rare species. Independent variables were as follows: locality (BG, KM, KL, BO, VA, BU, for explanation of symbols, see Table 1), *Sphagnum* species and subgenus (*Acutifolia*, *Cuspidata*, *Rigida*, *Sphagnum*, *Subsecunda*), wetness (lawn, hummock, carpet), and trophic state (moderately rich, poor, intermediate), all taken as factors or dummies. Since elevation and locality were highly correlated, elevation was not considered in the analysis.

We first used canonical correspondence analysis (CCA) to determine if any of the independent variables (localities, wetness, trophic state, and *Sphagnum* subgenera), when treated together, explained the variation of mite communities (simple effects). Next, these were compared to conditional effects of the same independent variables to detect any correlation or collinearity among independent variables or factors. This eliminated variables that were redundant or explained very little, so further analyses (i.e., those presented in Figures 4, 5, 6) focused only on the significant variables that explained most of the variation of mite communities in the conditional effects space.

## 3 RESULTS

### 3.1 Abundance and richness of mites

**FIGURE 3:**
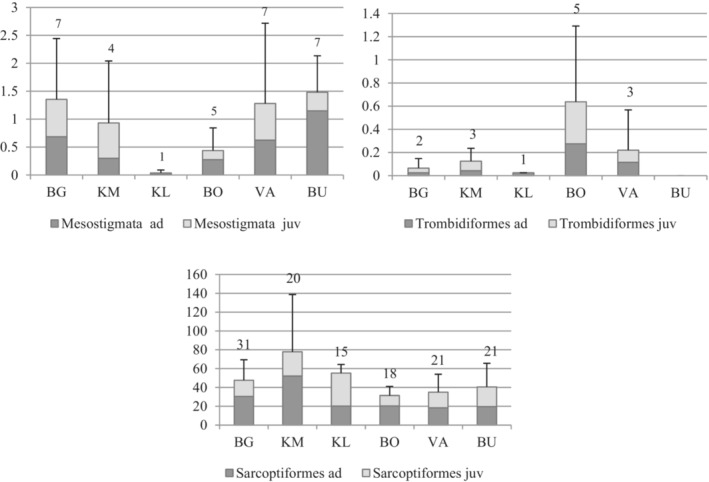
Average abundance (*A*, in 1000 ind./m^2^) (bars) of mites with standard deviation (whiskers), and average number of species (above bars) in mires in western Norway; the proportion of adults (ad) and juveniles (juv) is marked with bars of different shades; detailed information about the sampling locations is in Table 1.

In total, 59,777 specimens of mites were extracted from 27 *Sphagnum* samples. Sarcoptiformes were most abundant, constituting 97.86% of all mites. Mesostigmata and Trombidiformes represented 1.87% and 0.27% of mites, respectively. The highest total abundance of Sarcoptiformes was recorded at the location KM, while Mesostigmata were most abundant at BU, and Trombidiformes at BO (Figure 3); however, due to large variations among the samples, none of these differences were significant. In Mesostigmata and Trombidiformes, the juvenile instars were more abundant in extracted samples than adults, constituting an average of 51% and 57% of these groups, respectively. In Sarcoptiformes, the juveniles were less abundant than adults, representing an average of 38% of this group. The percentage of juvenile Sarcoptiformes was higher (63%) in only one locality (KL).

Overall, 154 mite species belonging to 54 families were recorded in mires (Appendix S1). Among them, 33 species (representing 13 families) belonged to the order Mesostigmata, 24 species (9 families) belonged to Trombidiformes, and 97 species (31 families) belonged to Sarcoptiformes (among which 96 species belonged to the suborder Oribatida). Ten species (6% of the total number), including one Mesostigmata, six Trombidiformes, and three Sarcoptiformes, were represented only by juveniles.

Seventeen species new to Norway are reported here (Table 2)—including four Mesostigmata, 12 Trombidiformes, and one Sarcoptiformes. Some of these new species records [Mesostigmata: *Ololaelaps sellnicki* Bregietova et Koroleva, Trombidiformes: *Stigmaeus* sp. nov. I] were abundant and found in several localities. Four species that are new records were represented only by juveniles. Among the new Trombidiformes records, five species are new to science.

### 3.2 Factors affecting mite communities

**FIGURE 4:**
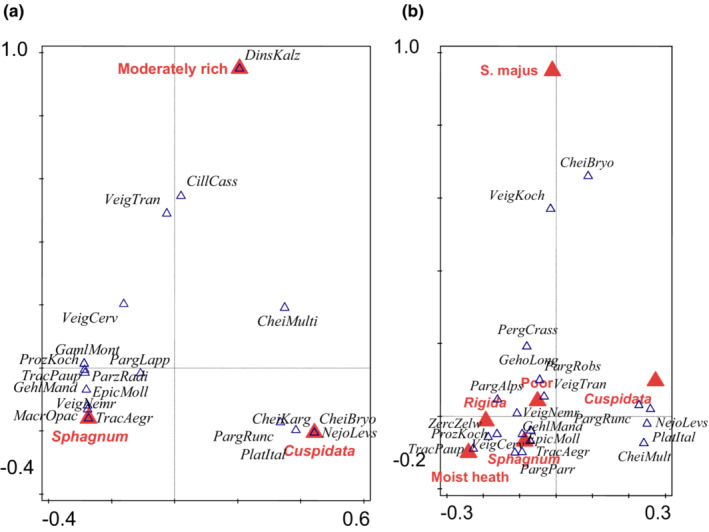
Results of canonical correspondence analysis (CCA) of Mesostigmata communities (represented by 20 best fitted species, represented by blue triangles, see Appendix S1 for species name abbreviations and environmental variables (see the Methods for the explanation)) in mires of western Norway, represented by red triangles. Names of species (or their abbreviations) are in Italic font. (a) Adults, total variation is 4.23084, explanatory variables account for 29.23% (adjusted explained variation is 18.62%). (b) Adults + juveniles, total variation is 3.15506, explanatory variables account for 52.91% (adjusted explained variation is 36.29%).

**FIGURE 5:**
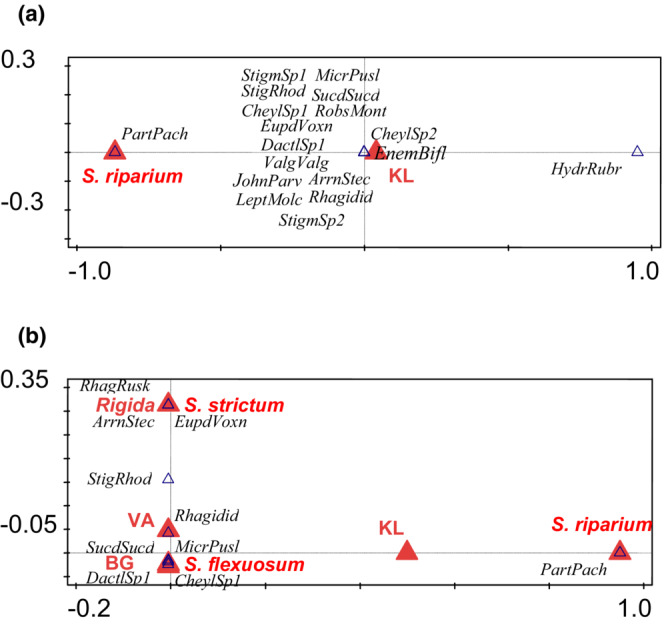
Results of canonical correspondence analysis (CCA) of Trombidiformes communities (represented by 10 best fitted species, represented by blue triangles, see Appendix S1 for abbreviations) and environmental variables (see the Methods for the explanation) in mires of western Norway, represented by red triangles. Names of species (or their abbreviations) are in Italic font. (a) Adults, total variation is 8.47626, explanatory variables account for 23.60% (adjusted explained variation is 11.84%). (b) Adults + juveniles, total variation is 5.74344, explanatory variables account for 73.74% (adjusted explained variation is 57.99%).

**FIGURE 6:**
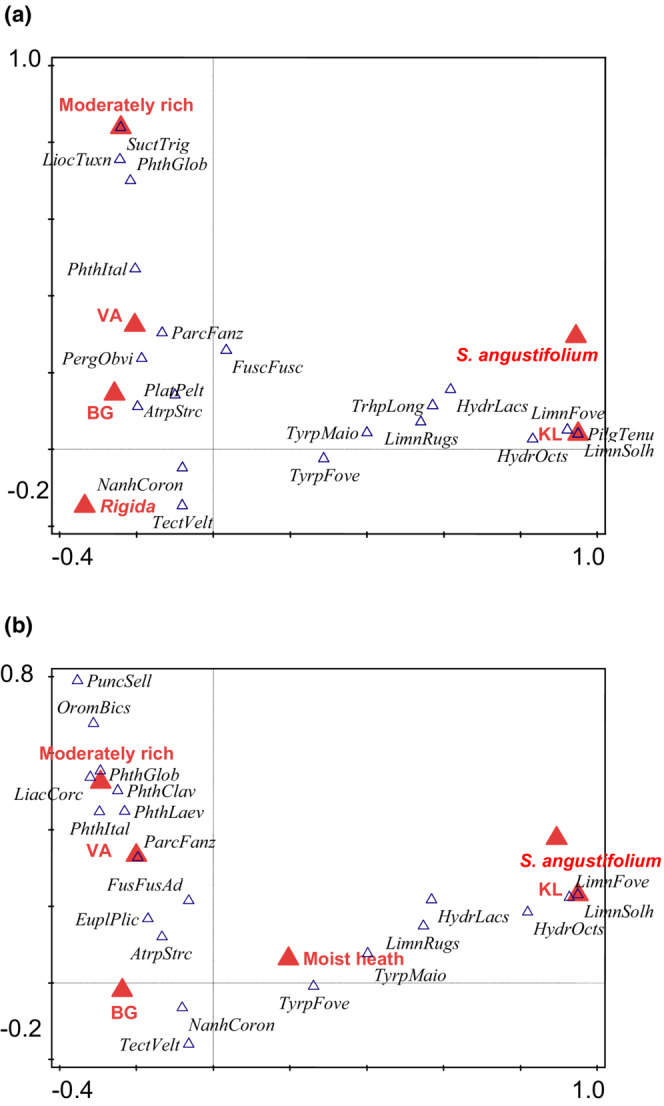
Results of canonical correspondence analysis (CCA) of Sarcoptiformes communities (represented by 20 best fitted species, represented by blue triangles, see Appendix S1 for abbreviations) and environmental variables (see the Methods for the explanation) in mires of western Norway, represented by red triangles. Names of species (or their abbreviations) are in Italic font. (a) Adults, total variation is 3.15835, explanatory variables account for 38.48% (adjusted explained variation is 20.03%). (b) Adults + juveniles, total variation is 2.90136, explanatory variables account for 39.26% (adjusted explained variation is 21.04%).

According to CCA, when all factors were considered together, they were not significant for mite orders; however, forward selection appeared significant for some factor levels, with the results differing between the ad only and ad+juv datasets (Table 3). When ad+juv were included, more levels of factors were significant (Figures 4, 5, 6, compare a vs. b).

**TABLE 3:** Forward selection results: conditional effects of independent variables at factor level in mite communities in mires of western Norway

Independent variable	Explains %	*p*
Mesostigmata – ad		
*Sphagnum* subgenus *Cuspidata*	14.2	.001
Trophy – Moderately rich	8.7	.007
*Sphagnum* subgenus *Sphagnum*	6.4	.032
Mesostigmata – ad+juv		
*Sphagnum* subgenus *Cuspidata*	13.1	.002
*Sphagnum* species *S. majus*	10.7	.005
Humidity – Moist heath	8.7	.007
*Sphagnum* subgenus *Sphagnum*	7.2	.007
Trophy – Poor	6.7	.016
*Sphagnum* subgenus *Rigida*	6.5	.037
Trombidiformes – ad		
Locality KL	11.8	.001
*Sphagnum* species *S. riparium*	11.8	.031
Trombidiformes – ad+juv		
*Sphagnum* species *S. riparium*	17.4	.001
Locality KL	17.4	.05
Locality VA	10.7	.02
*Sphagnum* subgenus *Rigida*	12.9	.007
*Sphagnum* species *S. strictum*	12.9	.014
Locality BG	8.5	.018
*Sphagnum* species *S. flexuosum*	6.8	.022
Sarcoptiformes – ad		
Locality KL	8.8	.004
*Sphagnum* species *S. angustifolium*	8.2	.026
Locality BG	5.8	.022
*Sphagnum* subgenus *Rigida*	5.5	.041
Locality VA	5.1	.045
Trophy – Moderately rich	5.1	.051
Sarcoptiformes – ad+juv		
Locality KL	10.0	.004
*Sphagnum* species *S. angustifolium*	8.1	.023
Locality BG	5.9	.02
Locality VA	5.2	.032
Trophy – Moderately rich	5.1	.046
Humidity – Moist heath	4.9	.053

Mesostigmata were affected by some levels of *Sphagnum* subgenus, *Sphagnum* species, and trophic level (Figure 4). Whether ad or ad+juv were considered, the most important factor was *Sphagnum* subgenus *Cuspidata*, which was distinguished from other *Sphagnum* subgenera by several characteristic mite species: *Nejordensia levis* (Oudemans et Voigts), *Platyseius italicus* (Berlese), *Cheiroseius bryophilus* Karg, *Ch. kargi* Gwiazdowicz, and *Paragamasus runciger* (Berlese). *Sphagnum* subgenus *Sphagnum* was also distinguished by a set of characteristic species. In contrast, the other factor levels differed depending on the data analyzed (ad vs. ad+juv) (Figure 4).

For Trombidiformes, including both adults and juveniles better explained the variation of this group (Figure 5). When only adults were considered, explanatory variables accounted for 23.60% of Trombidiformes variability, while with ad+juv, explanatory variables explained 73.74% of the variability of this order. The most important factor was locality KL (Table 3), with a number of characteristic species (Figure 5) [*Arrenurus stecki* Koenike, *Cheylostigmaeus* spp., *Dactyloscirus* sp., *Enemothrombium bifoliosum* (Canestrini), *Eupodes voxencollinus* Thor, *Hydryphantes ruber* De Geer, *Johnstoniana parva* Wendt, Wohltmann, Eggers et Otto, *Leptus molochinus* (C.L. Koch), *Microtrombidium pusillum* (Hermann), *Robustocheles montana* Zacharda, *Stigmaeus* spp., *Sucidothrombium sucidum* (L. Koch), *Valgothrombium valgum* (George)]. Another important factor was the *Sphagnum* species (*S. riparium*) with its characteristic species *Parathyas pachystoma* (Koenike).

Locality KL was also the most important factor for Sarcoptiformes (Table 3), with several aquatic Oribatida characteristics for this locality (Figure 6) [*Hydrozetes octosetosus* Willmann, *Limnozetes foveolatus* Willmann, *L*. *solhoyorum* Seniczak et Seniczak, and *Pilogalumna tenuiclava* (Berlese)]. Another important factor was *Sphagnum* species, that is, *S. angustifolium*, also characterized by aquatic Oribatida.

## 4 DISCUSSION AND CONCLUSIONS

This is the first study that demonstrates such a high diversity of mites in mires, highlighting the importance of these ecosystems for maintaining biodiversity. It is especially significant that mires host many unique species, which cannot be found in any other habitats. As many as 154 mite species were found with 20 *Sphagnum* species collected, including 33 species of Mesostigmata, 24 species of Trombidiformes, and 97 of Sarcoptiformes. In comparison, in other mires, the number of species found was lower, although Sarcoptiformes (or its suborder Oribatida) were always the most diverse group. For example, in northwestern Russia, 60 species of Sarcoptiformes, 15 species of Mesostigmata, and three species of Trombidiformes were found (Philippov et al., 2021), in Ireland, 43 species of Oribatida and 14 of Mesostigmata were recorded (Wisdom et al., 2011), and in southern Germany, 52 species of Oribatida were noted (Lehmitz et al., 2020). In the latter study, the discovered species richness of Oribatida was higher than that of vegetation and beetles, but lower compared to the richness of spiders (Lehmitz et al., 2020). Lower numbers of species found in the above‐mentioned studies can certainly be related to different sampling efforts or extraction efficiency (Minor et al., 2016), but the fact that only adults were identified to the species level is also of importance.

This study showed clearly that including juveniles is important for discovering mite diversity in mires, thus supporting our first hypothesis. Ten species (one Mesostigmata, six Trombidiformes, and three Sarcoptiformes) were represented only by juveniles. Furthermore, among 17 new species records for Norway, four species (all Trombidiformes) were represented in the samples only by the juvenile instars.

The high diversity of mites in Norwegian mires can also result from the fact that these mires are well preserved (Tanneberger et al., 2017) and are rich in diverse *Sphagnum* mosses (Flatberg, 2013). Peatlands dominated by *Sphagnum* host significantly richer mite fauna than, for example, those dominated by *Carex* (Barreto & Lindo, 2021). Habitat complexity is undoubtedly one of the most important factors in structuring biotic assemblages (Kovalenko et al., 2012). Different peat mosses vary considerably in their photosynthetic capacity (i.e., the maximum rate at which leaves can fix carbon during photosynthesis), productivity (Breeuwer et al., 2008), decomposition rate (Limpens & Berendse, 2003), peat accumulation, litter quality (Bengtsson et al., 2016), desiccation tolerance, and recovery ability (Hajek & Vicherová, 2014; Rydin, 1986, 1993). Different *Sphagnum* species also differ in the structure of their microbial communities (Bragina et al., 2012; Opelt et al., 2007). These differences are likely to influence the communities of other organisms inhabiting peat mosses, such as mites.

Oribatida are the most well‐studied mite group in mires, with more than 400 species recorded in the Holarctic region (Mumladze et al., 2013). Peat mosses provide a variety of foods to different feeding groups of Oribatida. Some oribatid species feed directly on *Sphagnum* tissue, contrary to the belief that peat mosses are not eaten by any herbivores (Rydin & Jeglum, 2006), but most species feed on associated algae, fungi, bacteria, protozoa, and nematodes (Lehmitz & Maraun, 2016). Oribatid mites can live in different parts of *Sphagnum*: on drier apical parts; in more basal parts; or in the spaces between *Sphagnum* leaves. Oribatida are closely dependent on microhabitat conditions, most notably the moisture level (Lehmitz et al., 2020) or the genus of *Sphagnum* present (Minor et al., 2016; Seniczak, Seniczak, Iturrondobeitia, et al., 2020; Seniczak, Seniczak, Schwarzfeld, et al., 2020), but also water chemistry (Seniczak et al., 2022). Therefore, any changes in peatlands, either due to climate change or human activities that affect water level or water chemistry, have strong impacts on the oribatid communities (Lehmitz et al., 2020; Markkula 1981, 1982; Seniczak et al., 2016; Seniczak et al., 2022). Dramatic changes in oribatid communities can be observed very quickly, even between different seasons (Seniczak, Seniczak, Graczyk, et al., 2019), so Oribatida seem to be very good bioindicators for short‐term changes in peatlands (Lehmitz et al., 2020; Seniczak et al., 2022).

Mesostigmata are less investigated in mires than Oribatida. They have been studied, for example, in Poland and Latvia, including both juvenile and adult forms, and although the number of species varied between the types of mires (10–35), several rare species new to these countries have been found. Mire communities include habitat‐specific Mesostigmata species that makes these habitats particularly important in terms of biodiversity (Kaczmarek et al., 2006, 2011; Kaczmarek & Marquardt, 2007, 2008; Marquardt & Kaczmarek, 2009; Salmane, 2006, 2009; Salmane & Spuņģis, 2015; Skorupski et al., 2008). The bioindicative reaction of Mesostigmata is less pronounced, that is, in contrast to Oribatida, none of the environmental variables were significantly associated with variation in the Mesostigmata communities, but the Mesostigmata results often support those of Oribatida (e.g., high dominance of aquatic species or a shift to generalists is often observed in both groups) (Seniczak et al., 2022).

Trombidiformes (except for water mites) are almost unknown from mires. Although these mites have been included in some studies, they were treated as a whole (e.g., Laiho et al., 2001; Seniczak, Seniczak, Graczyk, et al., 2019), without resolution to the species level. One of the trombidiform groups important in mires is the cohort Parasitengonina which comprises two main ecological groups: water mites (Hydrachnidia) and terrestrial Parasitengonina. In Europe, there have been a large number of studies on water mites inhabiting peatlands (e.g., Smit & van der Hammen, 1996; Stolbov et al., 2018; Więcek et al., 2012, 2013a, 2013b), and water mites seem to be represented in these habitats by relatively few species. For example, in Canada, where more than 500 species of water mite species are known, only about 30 were found in peatlands (Smith, 1987). In our study, we only collected three species of Hydrachnidia, but the methodological approach used (extraction of peat mosses) is not sufficient to discover the diversity of this group (Stryjecki et al., 2017; Więcek, Martin, & Gąbka, 2013).

Terrestrial Parasitengonina from mires have been studied to a far lesser extent, compared to water mites, and only 25 species are known from this type of habitat in Europe (Franke, 1942; Gabryś, 1996, 1997; Gabryś & Mąkol, 1994; Mąkol, 2005; Mąkol & Gulvik, 2002; Stålstedt et al., 2019; Willmann, 1939). In our study, this group was represented by seven species, including one new to Norway. *Johnstoniana parva* and *Valgothrombium valgum* are confined to amphibious biotopes; *Calyptostoma velutinum* and *Enemothrombium bifoliosum* are usually abundant in such biotopes; *Microtrombidium pusillum* and *Sucidothrombium sucidum* are often found in temporarily flooded areas; whereas the only representative of Erythraeidae recorded here, *Leptus molochinus*, has been known from a wider scope of habitats, including temporarily inundated ones (Stålstedt et al., 2019; Wohltmann et al., 2006). It is noteworthy that the larvae of *Calyptostoma velutinum* parasitize Tipulidae, and larvae of *Valgothrombium valgum* have been recorded as parasites of Ceratopogonidae, whereas the postlarval instars of three out of seven species recorded in the present study (*Calyptostoma velutinum*, *Enemothrombium bifoliosum,* and *Microtrombidium pusillum*) may serve as hosts for larvae of *Johstoniana parva* (Felska et al., 2018; Wohltmann et al., 2006).

The remaining terrestrial Trombidiformes, along with Endeostigmata (Sarcoptiformes), form the most heterogeneous group of mites and are found in a wide variety of environments. It is, therefore, difficult to characterize these mites as a whole, regarding their ontogeny, biology, ecology, and habitat preferences. They can be found in extremely different climatic zones and habitats, from very moist to very dry (Walter, 2009; Walter et al., 2009). However, the specimens collected in this study were primarily predators (Rhagidiidae, Cunaxidae, Stigmaeidae), with only *Eupodes* and *Bimichaelia* likely being fungivorous or omnivorous, respectively. This may be because the harshness of the mire environment filtered out some of the non‐predatory taxa typically seen in soil environments; however, more detailed comparisons with terrestrial environments would be needed to test this hypothesis. While most of the taxa recorded here are not recognized as typical and exclusive residents of *Sphagnum* (A. Kaźmierski, unpublished data), there may be some taxa that are mire‐ or wetland‐associated, where this information is simply unknown, due to their undescribed status or lack of detailed ecological study. For example, while the family Cunaxidae as a whole is more common in dry environments, the genus *Dactyloscirus* (the only cunaxid collected in this study) tends to be most commonly collected in wet environments such as bogs, wetlands, stream edges (M. Schwarzfeld, unpublished data).

Even though Trombidiformes were the least numerous group in the mires studied, their inclusion significantly increased our knowledge about the biodiversity of mires. Of the 17 species newly recorded for Norway, most were Trombidiformes (12). In addition, five of these species are considered new to science. These findings confirm that mires are fascinating and undiscovered habitats, and even in the relatively well‐studied northern hemisphere new species of mites can be discovered (Barreto & Lindo, 2021; Seniczak & Seniczak, 2009a, 2020, 2021).

Including the juvenile stages in the analyses was also very important in allowing us to better explain the variability of mite communities, what agreed with our second hypothesis. The variability of Mesostigmata was best explained by *Sphagnum* subgenus *Cuspidata*. Among mite species, characteristic of this subgenus was *Platyseius italicus*, an aquatic species often found in peatlands, both in bogs and fens, but also reported from other submerged habitats like streams and different water reservoirs (Bolger, Arroyo, & Piotrowska, 2018; Kaczmarek et al., 2006, 2011; Kaczmarek & Marquardt, 2007, 2008; Marquardt & Kaczmarek, 2009). Another species characteristic of subgenus *Cuspidata* was *Cheiroseius bryophilus*, a peatland specialist (Philippov et al., 2021; Salmane & Brumelis, 2010; Salmane & Spuņģis, 2015). Other species of this genus also prefer high levels of humidity, including *Ch. mutilus* (Berlese), which was found in the present study and was previously recorded from peatlands in Poland and Latvia (Kaczmarek et al., 2011; Kaczmarek & Marquardt, 2008; Salmane & Spuņģis, 2015; Skorupski et al., 2008). In contrast, *Ch. kargi*, also reported in the present study, has so far only been found in the litter of oak‐hornbeam forests in Poland (Gwiazdowicz, 2007). *Nejordensia levis* has been found in many different habitats, including in peatlands (Gwiazdowicz, 2007; Kaczmarek et al., 2011; Kaczmarek & Marquardt, 2008). Another species with a wide range of ecological tolerance is *Paragamasus runciger*, which is a forest species; however, it is also found (in low abundances) in peatlands (Kaczmarek & Marquardt, 2008). Interestingly, the members of the subgenus *Sphagnum* hosted a larger set of characteristic species. This *Sphagnum* subgenus is also known to host more diverse communities of microorganisms (Bragina et al., 2012; Opelt et al., 2007) and Oribatida (Minor et al., 2016; Seniczak, Seniczak, Iturrondobeitia, et al., 2020 ) than subgenus *Cuspidata*.

With the juvenile instars included, more environmental factors explained the variability of Mesostigmata communities. Additionally, different results regarding the effect of the trophic level were obtained. When only adults were analyzed, *Dinychus kaluzi* Mašán (only present in the extracted samples as an adult) distinguished moderately rich habitats. When juveniles were added, poor habitat was distinguished from the others by *Paragamasus robustus* (Oudemans) and *Veigaia transisalae* (Oudemans) (juveniles of these species were more abundant than adults). *Veigaia transisalae* has also frequently been found in peatlands in Poland and Latvia (Kaczmarek & Marquardt, 2008; Salmane & Spuņģis, 2015).

For Trombidiformes, including both adults and juveniles was even more important for explaining the variability of this group (explanatory variables account for 23.60% for ad and 73.74% for ad+juv). The most important factor was locality KL, with several characteristic species, including the water mites *Arrenurus stecki* and *Hydryphantes ruber*. *Arrenurus stecki* is characteristic of acidic waters and very often occurs in semi‐aquatic habitats connected with *Sphagnum* (Gerecke et al., 2016). *Hydryphantes ruber* is characteristic of vernal, astatic waters, and occurs in permanent pools and in lakes (Di Sabatino et al., 2010). Among the terrestrial Parasitengonina that distinguished this locality were *Sucidothrombium sucidum* and *Valgothrombium valgum*. The former species, common in northern Europe, is often found in moist habitats, including mires (Stålstedt et al., 2019). The latter species, confined to biotopes with regular inundations, has been also found in mires (e.g., Stålstedt et al., 2019; Willmann, 1933; Wohltmann et al., 2006). *Johnstoniana parva* is confined to amphibious (limnic) biotopes, *Enemothrombium bifoliosum* (Canestrini) is abundant in inundated areas, whereas *Microtrombidium pusillum* (Hermann) and *Leptus molochinus* (C.L. Koch) have been often found in areas with temporarily flooded soils (Łaydanowicz & Mąkol, 2008; Wohltmann et al., 2006). Another important factor was the *Sphagnum* species (*S. riparium*) with its characteristic species *Parathyas pachystoma*. This water mite is characteristic of vernal, astatic waters, and occurs in semi‐aquatic habitats (quagfens, swamps) as well (Di Sabatino et al., 2010).

Locality KL was also the most important factor for Sarcoptiformes, with several aquatic Oribatida distinguishing this locality. *Hydrozetes* and *Limnozetes* encompass truly aquatic species (Schatz & Behan‐Pelletier, 2008) and *Pilogalumna tenuiclava* is characteristic of moist mire habitats (Seniczak & Seniczak, 2022). Another important factor was *Sphagnum* species, that is, *S. angustifolium* was also characterized by aquatic Oribatida.

Regardless of the undisputed impact on the assessment of biodiversity, the inclusion of juvenile stages in the research also significantly contributes to the understanding of the biology and life cycle of species whose taxonomy and systematics are based on adult stages. This is of particular importance in the case of species with a specific phenology, for example, with the appearance of juvenile stages limited to short periods throughout the year. For the identification of juvenile stages, in the case of species whose identification is based on morphological features, molecular analysis may be extremely helpful.

In summary, contrary to the established convention that peatlands are species‐poor habitats, well‐preserved mires like the ones studied here hide significant diversity treasures. In exploring peatlands, it is important to include often ignored mite groups, for example, within order Trombidiformes, as well as taking into account the often‐overlooked juvenile stages.

## AUTHOR CONTRIBUTIONS

**Anna Beata Seniczak:** Conceptualization (equal); data curation (equal); formal analysis (equal); investigation (equal); methodology (equal); project administration (equal); validation (equal); visualization (equal); writing – original draft (equal); writing – review and editing (equal). **Stanisław Seniczak:** Conceptualization (equal); data curation (equal); formal analysis (equal); investigation (equal); methodology (equal); validation (equal); writing – original draft (equal). **J. Carlos Iturrondobeitia:** Conceptualization (equal); formal analysis (equal); validation (equal); visualization (equal); writing – original draft (equal). **Martyna Marciniak:** Data curation (equal); investigation (equal); validation (equal); writing – original draft (equal). **Sławomir Kaczmarek:** Data curation (equal); funding acquisition (equal); investigation (equal); validation (equal); writing – original draft (equal). **Joanna Mąkol:** Data curation (equal); investigation (equal); validation (equal); writing – original draft (equal). **Andrzej Kaźmierski:** Data curation (equal); investigation (equal); validation (equal); writing – original draft (equal). **Andrzej Zawal:** Investigation (equal); validation (equal); writing – original draft (equal). **Marla Schwarzfeld:** Validation (equal); writing – original draft (equal); writing – review and editing (equal). **Kjell Ivar Flatberg:** Conceptualization (equal); data curation (equal); investigation (equal); methodology (equal); validation (equal); writing – original draft (equal).

## Supporting information


Appendix S1

## Data Availability

The data that supports the findings of this study are available in the supplementary material of this article.
